# LncRNA PPM1A-AS Regulate Tumor Development Through Multiple Signal Pathways in T-Cell Acute Lymphoblastic Leukemia

**DOI:** 10.3389/fonc.2021.761205

**Published:** 2021-10-21

**Authors:** Guoli Li, Xinyue Lei, Yingchi Zhang, Zhe Liu, Kegan Zhu

**Affiliations:** ^1^ Department of Immunology, Biochemistry and Molecular Biology, 2011 Collaborative Innovation Center of Tianjin for Medical Epigenetics, Tianjin Key Laboratory of Medical Epigenetics, Tianjin Medical University, Tianjin, China; ^2^ State Key Laboratory of Experimental Hematology, National Clinical Research Center for Blood Diseases, Institute of Hematology & Blood Diseases Hospital, Chinese Academy of Medical Sciences & Peking Union Medical College, Tianjin, China; ^3^ Key Laboratory of Immune Microenvironment and Disease of the Ministry of Education, Tianjin Medical University, Tianjin, China; ^4^ Tianjin Key Laboratory of Radiation Medicine and Molecular Nuclear Medicine, Institute of Radiation Medicine, Tianjin, China

**Keywords:** PPM1A-AS, T-ALL, Notch4, STAT3, Akt

## Abstract

ALL (Acute lymphoblastic leukemia) is the most common pediatric malignancy and T-ALL (T-cell acute lymphoblastic leukemia) comprises about 15% cases. Compared with B-ALL (B-cell acute lymphoblastic leukemia), the prognosis of T-ALL is poorer, the chemotherapy is easier to fail and the relapse rate is higher. Previous studies mainly focused in Notch1-related long non-coding RNAs (lncRNAs) in T-ALL. Here, we intend to investigate lncRNAs involved in T-ALL covering different subtypes. The lncRNA PPM1A-AS was screened out for its significant up-regulation in 10 T-ALL samples of different subtypes than healthy human thymus extracts. Besides, the PPM1A-AS expression levels in 3 T-ALL cell lines are markedly higher than that in CD45^+^ T cells of healthy human. We further demonstrate that PPM1A-AS can promote cell proliferation and inhibit cell apoptosis *in vitro* and can influence T-ALL growth *in vivo*. Finally, we verified that PPM1A-AS can regulate core proteins, Notch4, STAT3 and Akt, of 3 important signaling pathways related to T-ALL. These results confirm that lncRNA PPM1A-AS can act as an oncogene in T-ALL and maybe a potential clinical target of patients resistant to current chemotherapy or relapsed cases.

## Introduction

T-cell acute lymphoblastic leukemia (T-ALL) is a hematologic malignancy induced by the transformation of T-cell progenitors (1). The prominent feature of T-ALL is the uncontrolled proliferation of immature T lymphocyte, frequent large thymic masses, enlarged spleen and pleural effusions (1, 2). T-ALL accounts for 15% of childhood and 25% of adult ALL cases and childhood acute lymphoblastic leukemia (cALL) causes the most frequent death from cancer in pediatrics (3). So far, genetic alterations, including point mutations, chromosomal rearrangements and the loss or gain of chromosome, have been well studied due to the development of genome-wide sequencing. Dozens of oncogenes or tumor suppressors are found to be dys-regulated in T-ALL. As a result, modern combined chemotherapy remarkably raised the overall survive rate in patients especially pediatric patients with T-ALL. Although T-ALL cases are divided into different subgroups characterized by one particular transcription factor which is ectopic expressed, for example TLX1、TLX3、LMO、HOXA and so on, but almost T-ALL cases owe not only one biologically relevant genomic lesion (4). In some cases, more than 10 mutated genes work together and thus lead to the transformation of T cells into aggressive leukemia cells with enhanced proliferation and survival characteristics, impaired differentiation, altered cell cycle and metabolism properties (5). In fact, there are more than 100 genes were found abnormal in T-ALL (5). The screening out of novel molecules that participate in several regulating pathways may contribute to the clinical treatment of complex T-ALL cases which are resistant to given drugs or relapsed cases.

LncRNAs are a new class of RNAs which is more than 200 nucleotides in size with no defined open reading frames. LncRNAs are usually much lower expressed than mRNAs but can be specially expressed in particular tissue. LncRNAs are found to play roles in many normal life processes, such as neuroregulation, spermatogenesis, muscle regeneration and erythropoiesis (6). Besides, ectopic expression of lncRNA is closely related to different diseases including cancers (6). The latest researches show that lncRNAs participate in tumorigenesis, tumor proliferation, migration, invasion and metabolism (7). Thus, lncRNAs may be used as diagnostic markers, novel therapeutic targets and potential prognostic markers in cancers. In leukemia, lncRNAs also have important functions (7). For example, in BLL (B-cell lymphoblastic leukemia), lncRNA GAS5 was proved to regulate metastasis by repressing miR-222 (8) and lncRNA ZEB-AS1 could influence tumor development by targeting IL11/STAT3 signal pathway (9). Besides, Trimarchi et al. focused in Notch-regulated lncRNAs while Takaomi Sanda et al. focused in TAL1 complex-regulated lncRNAs in T-ALL (10, 11). Furthermore, another lncRNA, NALT, was also involved in T-ALL development by inducing Notch1 activation (12). Despite of these findings, more efforts are needed to make the lncRNA regulatory network in leukemia clearer.

In the current study, we found a new lncRNA, which we named as PPM1A-AS, was overexpressed in patients with T-ALL at the first time. Then, we demonstrated that T-ALL tumor cell proliferation ability was closely connected to the PPM1A-AS expression level. Moreover, PPM1A-AS could influence the tumor cell apoptosis *in vitro*. Next, we established tumor model in NOD-SCID mice and demonstrated that PPM1A-AS could also promote T-ALL development *in vivo*. Finally, we performed whole-transcriptome deep sequencing in wild- or PPM1A-AS-knockdown-Jurkat cells. Compared to the wild group, we detected 288 up expressed genes and 313 down expressed genes in PPM1A-AS-knockdown group. By KEGG pathway analysis, the differentially expressed genes are enriched in Notch signal pathway and PI3K-Akt signaling pathway, which take important roles in T-ALL tumor development. We then verified that phosphorylated Akt, phosphorylated STAT3 and Notch4 protein levels are positive related with PPM1A-AS. To conclude, we find that PPM1A-AS can work as an oncogene and can regulate several pathways in T-ALL, and thus may be provided as a potential clinic target for T-ALL patients with multiple gene mutations.

## Materials and Methods

### Cell Culture

The T-ALL cell lines Jurkat, CEM and MOLT4 were cultured in RPMI1640 (Gibco, NY, USA) with 10% fetal bovine serum (FBS). They were maintained at 37°C in a humidified incubator with 5% CO2.

### Human CD45^+^ T Cell Isolation

The peripheral blood were collected from healthy human and red blood cell lysis was conducted with lysis buffer (Solarbio, Beijing, China). The CD45^+^ T cells were isolated with magnetic beads (Stemcell, Vancouver, Canada) according to the manufacturer’s instructions.

### Quantitative Real Time PCR

Total RNA of T-ALL cells was extracted with TRIzol Reagent (Thermo Fisher Scientific, MA, USA) and 1ug total RNA was reverse-transcribed into complementary DNA(cDNA) using the RevertAid First Strand cDNA Synthesis Kit (Thermo). Quantitative real-time PCR was performed to determine the RNA expression using SYBR Green master mix (DBI^®^Bioscience, Ludwigshafen, German) with specific primers listed below. All of the reactions were run in triplicate and the relative levels of lncRNA were normalized to 18S rRNA. The sequences of the primers were as follows: hGAPDH Forward: 5’- CTTTTGCGTCGCCAGCCGAG -3’; hGAPDH Reverse: 5’- CCAGGCGCCCAATACGACCA -3’; PPM1A-AS Forward: 5’- AGTCCTGGACAGTCTTTAGGC -3’; PPM1A-AS Reverse: 5’- AGGTGTGTGCTGGGAAATGT -3’.

### Cell Nucleus/Cytoplasm Fraction Isolation

Cytoplasmic and nuclear RNA were isolated and purified using the PARIS™ Kit (ThermoFisher, #AM1921) according to the manufacturer’s instructions.

### Knockdown and Overexpression

For knockdown assay, oligos encoding shRNA specific for PPM1A-AS were ligated into pSUPER.retro.puro, and the fragment containing the H1 promoter and hairpin sequences was subcloned into the lentiviral shuttle pCCL.PPT.hPGK.GFP.Wpre. The shRNA target sequences were as follows: shPPM1A-AS-1, GCATCAAGAAGAACAGCTA; shPPM1A-AS-2, GGTTGATCTGTGCGGCAAA. For overexpression assay, lncRNA PPM1A-AS sequence was ligated into the lentiviral shuttle pCCL.PPT.hPGK.IRES.GFP/pre. These plasmids were used to produce lentivirus in HEK293T cells with the packaging plasmids pMD2.BSBG, pMDLg/pRRE and pRSV-REV. Cells were infected with lentivirus and sorted by GFP signal to generate a stable cell line.

### Cell Counting Kit-8 Assay

To assess cell proliferation ability, Jurkat, CEM and MOTL4 cells infected with control lentivirus, lncRNA PPM1A-AS-knockdown lentivirus or lncRNA PPM1A-AS-overexprssion lentivirus were seeded into 96-well plates at a density of 2000 cells per well. At 0, 24, 48, 72, 96, 120 hours after the cells were seeded, CCK-8 reagent (Dojingdo, Japan) was mixed with the cells for 1h incubation at 37°C. The absorbance value was measured at 450nm with Microplate reader.

### EdU Assay

The EdU assay was conducted with Cell-Light EdU Apollo567 In Vitro Kit (RiboBio Co., Guangzhou, China) according to the manufacturer’s instructions of suspension cell. Briefly, Jurkat, CEM and MOTL4 cells were infected with control, lncRNA PPM1A-AS-knockdown or lncRNA PPM1A-AS-overexprssion lentivirus and then 5000 cells of each group were planted in 24-well plates. 24 hours later, the cells were remarked with EdU and made smears. After cell fixation and Apollo staining, the slides were observed and took photos under a microscope at 100×magnification.

### Cell Apoptosis Assay

Jurkat and CEM cells infected with control lentivirus, lncRNA PPM1A-AS-knockdown lentivirus or lncRNA PPM1A-AS-overexprssion lentivirus were collected and incubated with annexin V and PIfor 15min. The apoptotic cells were detected by BD flow cytometer. The annexin positive but PI negative staining indicated the early apoptotic cells. The annexin V and PI both positive staining indicated cells in necrosis (post-apoptotic necrosis or late apoptosis). The proportion of total apoptotic cells are the sum of these two parts of cells.

### Western Blotting

The control or infected cells were rinsed with PBS and lysed in RIPA Lysisbuffer (Beyotime, China) supplemented with Protease and Phosphatase Inhibitor (Cell Signaling Technology Inc., USA) on ice for 30min. The cell lysates were centrifuged for 10min (12000 g, 4°C) and the supernatant was collected. The protein concentration was calculated with Pierce BCA protein assay kit (Thermo) and equivalent quantities of protein were separated on 10% SDS-PAGE gels. Then the proteins were transferred onto a nitrocellulose membrane (Bio-Rad, CA, USA). After blocking with 5% non-fat milk at room temperature for 1h, the membranes were immunostained with primary antibodies at 4°C overnight, washed three times in TBST, and then incubated with secondary antibody at room temperature for 1h. Finally, the protein bands on membranes were detected with an enhanced chemiluminescence reagent (Millipore) and captured using a luminescence instrument (Tanon, Shanghai, China). The gray density of protein bands was determined by Image J software. The primary antibodies were listed below: Akt (#9272, Cell Signaling Technology); T308-pAkt (#13038, Cell Signaling Technology), S473-pAkt (#4060, Cell Signaling Technology); STAT-3(#9132, Cell Signaling Technology), T705-pSTAT3 (#9131, Cell Signaling Technology), S727-pSTAT3 (#9134, Cell Signaling Technology); Notch4 (ab184742, Abcam).

### T-ALL Xenograft Model

Female NOD-SCID mice (4-6 weeks old) were purchased from the Model Animal Research Center of Nanjing University (Nanjing, China) and housed under pathogen-free conditions in Tianjin Medical University. Jurkat cells were infected with control lentivirus or lncRNA PPM1A-AS-knockdown lentivirus (2×10^6^), suspended in 200ul PBS and then injected into the mice by tail intravenous injection. After 45 days later, the peripheral blood was taken from mice, the mice were sacrificed, the spleens were photographed and weighed and the bone marrow were dissected. The animal study was reviewed and approved by Tianjin Medical University Animal Care and Use Committee.

### RNA Sequencing

RNA was isolated from Jurkat cells injected with control lentivirus or lncRNAPPM1A-AS-knockdownlentivirus using Trizol (Invitrogen) following manufacture instructions. Biological samples in duplicate were submitted to Novogene Co., Ltd for RNA sequencing. Barcoded sequence libraries were constructed using TruSeq RNA Sample Prep kit (Illumina), and sequenced on a HiSeq 2000 instrument.

### Statistical Analysis

Data analyses were undertaken with GraphPad PRISM 8.0. The results were shown as mean ± standard deviation (SD), from three independent experiments and analyzed *via* the Student’s t-test. The value p <0.05 was considered statistically significant.

## Results

### LncRNA PPM1A-AS Is Up-Regulated in T-ALL

**Figure 1:**
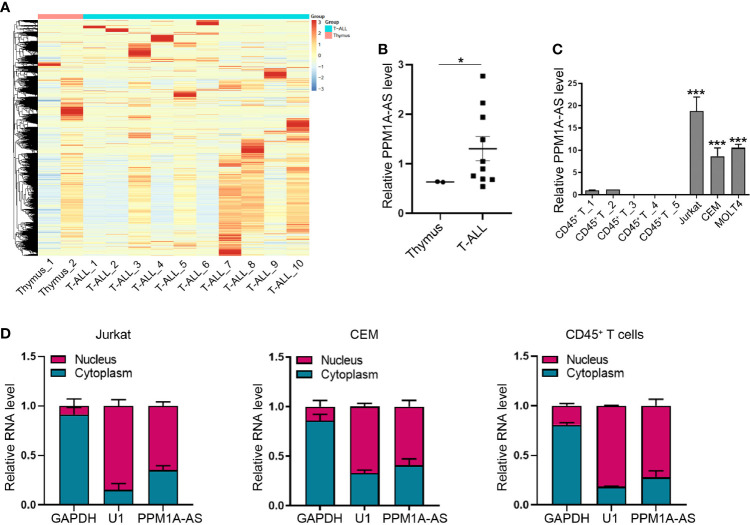
LncRNA PPM1A-AS is up expressed in T-ALL. **(A)** The heatmap of lncRNA expression in 10 T-ALL samples and 2 healthy human whole thymus extracts. **(B)** The relative expression level of lncRNA PPM1A-AS in 10 T-ALL samples and 2 healthy human thymus extracts. **(C)** RT-qPCR analysis of lncRNA PPM1A-AS in T-ALL cell lines and healthy human CD45^+^ T cells. **(D)** The nuclear and cytoplasmic distribution of lncRNA PPM1A-AS in Jurkat, CEM and T cells extracted from healthy human. Mean ± SD. *P < 0.05. ***P < 0.001.

To identify lncRNAs differentially expressed in T-ALL, we collected and analyzed RNA sequencing (RNA-seq) data of 10 T-ALL patients and 2 human whole thymus extracts from public database (GSE110636 (13), GSE57982 (10), the sample numbers were listed in **Supplementary Table 1**). The 10 T-ALL patients were belonging to different genetic subgroups. The heatmap was showed in **Figure 1A**. Among these lncRNAs, we found that CTD-2184C24.2 (ENST00000553775) was obviously increased in T-ALL patients (**Figure 1B**). CTD-2184C24.2 is a transcript antisense to *PPM1A*. So, we named this lncRNA as PPM1A-AS after this gene. Moreover, we tested the expression level of PPM1A-AS in T-ALL cell lines Jurkat, CCRF-CEM and MOLT4. The T cells extracted from healthy human blood with CD45^+^ magnetic beads were used as control. As a result, PPM1A-AS was significantly overexpressed in all three T-ALL cell lines than T cells extracted from healthy persons (**Figure 1C**). Finally, we verified the distribution of PPM1A-AS in T cells. No matter in normal or cancerous T cells, PPM1A-AS was existing in both nucleus and cytoplasm, mainly in nucleus (**Figure 1D**). These results also indicated that the PPM1A-AS had no shuttle between nucleus and cytoplasm during T-ALL formation.

### LncRNA PPM1A-AS Promotes Cell Proliferation and Inhibits Cell Apoptosis in T-ALL Cell Lines

**Figure 2:**
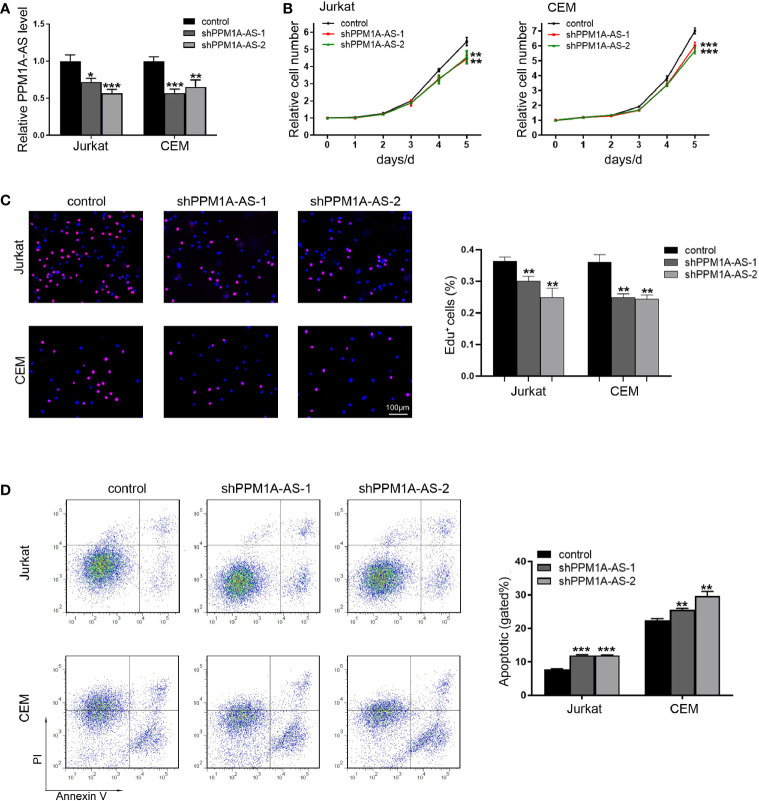
Knockdown of lncRNA PPM1A-AS can inhibit cell proliferation and promote cell apoptosis. **(A)** The efficiency of PPM1A-AS shRNAs in Jurkat and CEM cell lines. **(B)** CCK-8 analyses of the proliferation rates of Jurkat and CEM cells infected with control or PPM1A-AS-knockdown lentivirus. **(C)** EdU analyses of the proliferative ability of Jurkat and CEM cells infected with control or PPM1A-AS-knockdown lentivirus. Left panel: representative images; right panel: average percentage of EdU^+^ cells counted in each field. Scale bar, 100μm. **(D)** Flow cytometry analyses of T-ALL cell apoptosis after infected with control or PPM1A-AS-knockdown lentivirus. Left panel: representative images; right panel: percentage of apoptotic cells. Mean ± SD. *P < 0.05. **P < 0.01. ***P < 0.001.

**Figure 3:**
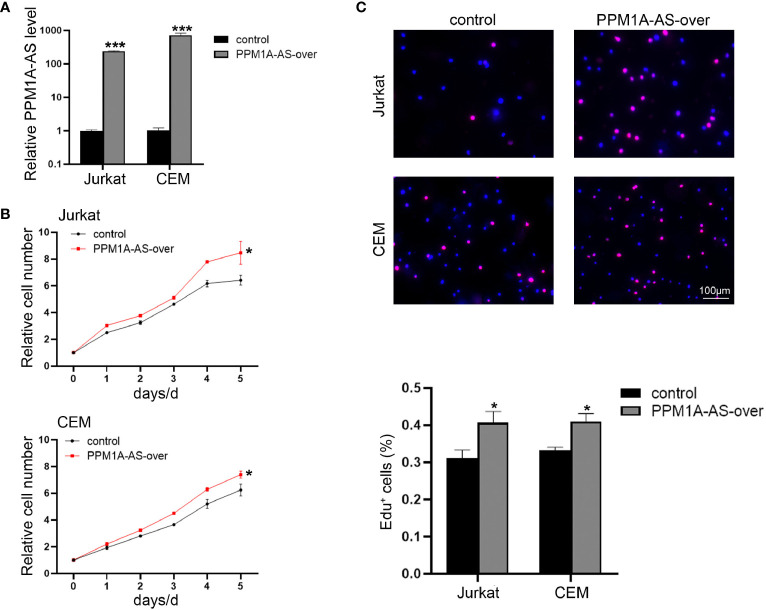
Overexpression of lncRNA PPM1A-AS can promote cell proliferation. **(A)** The efficiency of PPM1A-AS overexpression in Jurkat and CEM cell lines. **(B)** CCK-8 analyses of the proliferation rates of Jurkat and CEM cells infected with control or PPM1A-AS-overexpression lentivirus. **(C)** EdU analyses of the proliferative ability of Jurkat and CEM cells infected with control or PPM1A-overexpression lentivirus. Upper panel: representative images; bottom panel: average percentage of EdU^+^ cells counted in each field. Scale bar, 100μm. Mean ± SD. *P < 0.05. ***P < 0.001.

In order to explore the function of PPM1A-AS, stable clones with knockdown and overexpression of PPM1A-AS were generated with shRNA and plasmid *via* lentivirus technology in T-ALL cell lines, Jurkat, CEM and MOTL4. Firstly, we evaluated the efficiency of PPM1A-AS knockdown (**Figure 2A** and **Supplementary Figure 1A**) and overexpression by RT-qPCR (**Figure 3A** and **Supplementary Figure 1D**). The PPM1A-AS RNA level was about 25% lower by two different shRNAs and hundreds higher by overexpression vector. Then we performed CCK-8 and EdU assays to test the cell proliferation ability. Compared to the control group, cells infected with PPM1A-AS shRNA lentivirus showed significantly slower proliferation rates (**Figures 2B, C** and **Supplementary Figures 1B, C**) while overexpression of PPM1A-AS enhanced cell proliferation (**Figures 3B, C** and **Supplementary Figures 1E, F**). Besides, we examined cell apoptosis by flow cytometry. We found that knockdown of PPM1A-AS could induce T-ALL cell death (**Figure 2D**). Taken together, we conclude that lncRNA PPM1A-AS may have the potential to serve as an oncogene in T-ALL.

### LncRNA PPM1A-AS Regulates T-ALL Development *In Vivo*

**Figure 4:**
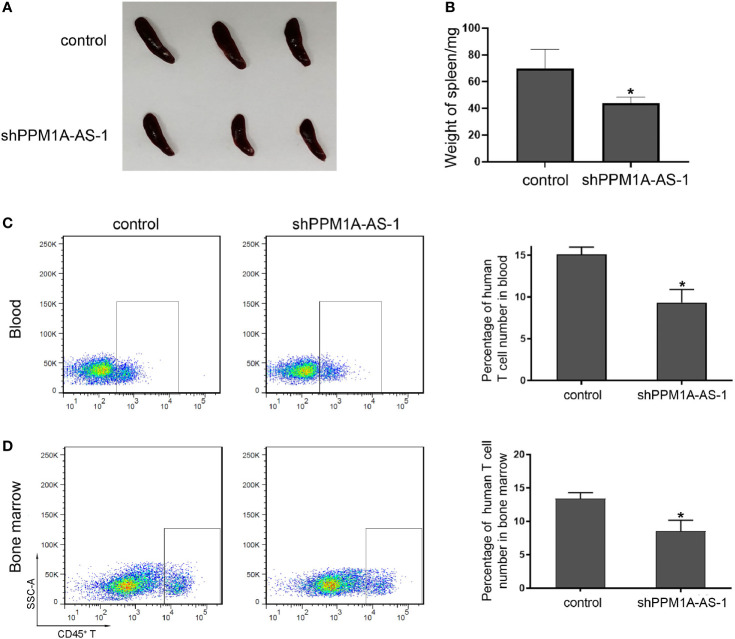
LncRNA PPM1A-AS can enhance tumor development in NOD-SCID mice. Jurkat cells were infected with control or PPM1A-AS-overexpression lentivirus and transplanted mice by tail intravenous injection. About 45 days later, the mice were sacrificed, the photos were taken and relative data were detected. **(A)** Representative images of spleens from mice. **(B)** Quantitative analyses of the weights of the spleens. **(C)** Flow cytometry analyses of human CD45^+^ T cells in peripheral blood of mice. Left panel: representative images; right panel: average percentage of CD45^+^ T cells in peripheral blood from each mouse. **(D)** Flow cytometry analyses of human CD45^+^ T cells in bone marrow of mice. Left panel: representative images; right panel: average percentage of CD45^+^ T cells in bone marrow from each mouse. Mean ± SD. *P < 0.05.

To further determine the role of lncRNA PPM1A-AS in T-ALL, we established tumor xenograft model in NOD-SCID mice. We cultured Jurkat cells infected with blank lentivirus or lncRNA PPM1A-AS-konckdown lentivirus and transplanted mice by tail intravenous injection. About 45 days later, we sacrificed the mice, photographed and measured the spleens, detected the proportion of tumor cells in peripheral blood and bone marrow. As the pictures showed in **Figure 4A**, the spleens from mice of the control group were much enlarged than the PPM1A-AS knockdown group. The statistical data were in **Figure 4B** and the spleens’ average weight of control mice was obviously larger. Moreover, the proportion of tumor cells in peripheral blood (**Figure 4C**) or bone marrow (**Figure 4D**) from control mice were much more than the mice of PPM1A-AS knockdown group, which means PPM1A-AS knockdown can repress T-ALL tumor development *in vivo*.

### LncRNA PPM1A-AS Target Genes in Multiple Signal Pathways

**Figure 5:**
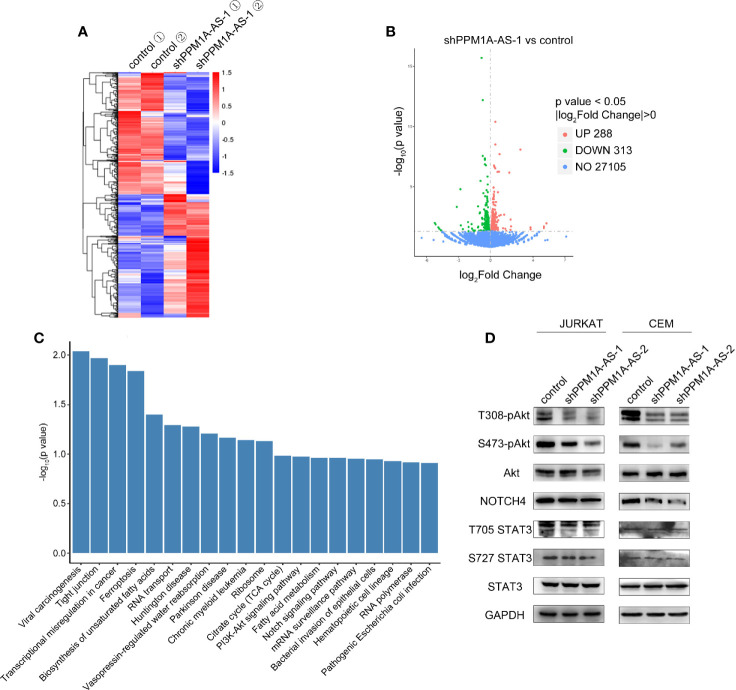
LncRNA PPM1A-AS regulates multiple signaling pathways in T-ALL. **(A)** The heatmap of transcriptome sequencing of Jurkat cells infected with control or PPM1A-AS-knockdown lentivirus. **(B)** The volcano plot representation of differentially expressed mRNAs upon PPM1A-AS knockdown. **(C)** Significantly enriched pathways relative to differentially expressed mRNAs upon PPM1A-AS knockdown. **(D)** Western blot analysis of core proteins belonging to T-ALL relative signaling pathways.

To further investigate the mechanism by which PPM1A-AS promotes T-ALL progression, we performed RNA-seq in Jurkat cells stably transduced with shRNAs targeting PPM1A-AS or a non-targeting control. Two biological replicates were included in each group and high correlations were observed between the replicates (**Figure 5A**). We then performed differential gene expression analysis, and identified 288 and 313 genes significantly upregulated and downregulated after knockdown of PPM1A-AS (**Figure 5B** and **Supplementary Table 2**). The major signal pathways that the differently expressed genes involved in were analyzed by KEGG database (**Figure 5C**). Among these pathways, Notch signaling pathway and PI3K-Akt signaling pathway are the major pathways participated in T-ALL tumorigenesis and development (14). We then tested if PPM1A-AS has any role in the well-known oncogenic pathways involved in T-ALL. *AKT*, *NOTCH* and *STAT* are core genes of PI3K-Akt signaling pathway, Notch signaling pathway and JAK-STAT3 signaling pathway respectively (15). We chose proteins of these genes and examined their expression after PPM1A-AS knockdown. The whole protein levels of STAT3 and Akt were not changed but the phosphorylated protein (T308, S473 of Akt and T705, S727 of STAT3) were significantly downregulated by PPM1A-AS knockdown (**Figure 5D**). Besides, the Notch4 (**Figure 5D**) but not Notch1 (data were not showed) protein level was also consistent with PPM1A-AS RNA level. Therefore, these data showed that PPM1A-AS could regulate multiple proteins and thus have roles in several T-ALL related pathways.

## Discussion

ALL is the most common pediatric malignancy and T-ALL comprises about 15% cases (16). Till now, scientists have discovered many oncogenic and tumor suppressor pathways that participate in T-ALL transformation and development. Notch signaling is an oncogenic pathway which is activated in more than 65% of T-ALL patients by activating Notch1 gene mutations (14, 17). Notch4 is also a member of NOTCH family of receptors but its role in T-ALL is not as clear as Notch1 (18). James et al. (2014) discovered that Notch4 did not signal in response to ligand but it could repress the signaling from Notch1 receptor. Notch4 could bind unprocessed, full-length Notch1 and altered the subcellular localization of Notch1 (19). Costa et al. found that genetic deletion of Notch4 did not result in an overt phenotype in mice as well as other publications. But the expression of Notch4 was required for tumor onset and early tumor perfusion in a mouse model of breast cancer, despite the phenomenon that the final tumor size was similar between tumors grown in wild type and Notch4-null hosts (20). Besides, PI3K-AKT signaling is also an important oncogenic signaling pathway in T-ALL (17). Transgenic overexpression of an active form of AKT in T cell progenitors results in increased PI3K signaling and induces T-ALL in mice (21). Moreover, there is another oncogenic signaling pathway, JAK-STAT signaling, playing roles in T-ALL (17). Approximately 10% of T-ALLs show gain-of-function mutations in IL7R and result in constitutive JAK-STAT signaling (22). In the research of 116 clinical cases, authors found that phosphorylated STAT3, but not pSTAT5 or pSTAT6, predicts better prognosis in the smoldering type of T-ALL (23). Due to these findings of oncogenic or tumor suppressed mechanism contributing to T-ALL, abundant chemotherapy protocols are drawn into clinical treatment and lead to a consequent gradual progress in cure rate. Despite this improvement, patients with primary resistant T-ALL or those with relapsed disease still faced terrible prognosis. So, we need more efforts to reveal other specific therapeutic targets underlying T-ALL development. In fact, many clinical T-ALL cases contain more than one gene mutation and may involve several signaling pathways which function in cancer transformation and grown (24). Combined application of chemotherapy drugs may be benefit to these complicated cases. Here, we discover that lncRNA PPM1A-AS can regulate Notch4, phosphorylated AKT, phosphorylated STAT3 expression in the same time and thus affect Notch signaling, PI3K-AKT signaling as well as JAK-STAT signaling pathways. This finding indicates the existence of lncRNA regulating different oncogenic or tumor suppressor pathways in T-ALL and may provide a new thought to solve complex clinical cases.

Due to the establishment of Sanger sequencing, the Human Genome Project was conducted in the worldwide which discovered that only 1.2% of the human genome represents protein coding exons and most genomic DNA is non-coding (25). The same phenomena were also verified in other eukaryotes and gave rise to heated debates in the scientific community about if they are transcriptional noises. The first long non-coding RNA H19 was found in the late 1980s, even though it was classified as an mRNA at that time (25). The function of lncRNA remained a mystery through a century until another lncRNA, Xist, was characterized to function in X-chromosome inactivation in mammals in the early 1990s (25). The rapid development of high-throughput sequencing technologies led to an explosion in the number of newly identified and uncharacterized lncRNAs. But many challenges in lncRNA biology remain, including accurate annotation, functional characterization and clinical relevance. Here, we focus on lncRNAs in T-ALL and tried to do our bit for the improvement of lncRNA regulatory network. We researched a new lncRNA, which we named as PPM1A-AS because it’s an antisense lncRNA of gene *PPM1A*, at the first time. We found PPM1A-AS was up-expressed in T-ALL patients and cell lines. By *in vitro* and *in vivo* assays, PPM1A-AS was proved to be benefit for T-ALL development through regulating cell proliferation and apoptosis. Next, we performed transcriptome sequencing using RNAs extracted from Jurkat cells which were infected with control or PPM1A-AS-knockdown lentivirus. KEGG pathway analysis revealed that PPM1A-AS was probably involved in Notch signaling and PI3K-Akt signaling pathways. We then tested if PPM1A-AS could influence expression of core proteins in these pathways by western blotting. The results showed that knockdown of PPM1A-AS didn’t affect the level of Notch1 and total protein of Akt but could decrease the phosphorylated Akt and Notch4 expression. Furthermore, we also detected lncRNA PPM1A-AS’s role in another oncogenic pathway, JAK-STAT signaling pathway. PPM1A-AS can impact phosphorylated STAT3 but not total STAT3 protein level. In conclusion, we not only make a break of functions of a new lncRNA but also make a contribution to the new roles of lncRNA in T-ALL. Finally, the particular mechanism which lncRNA PPM1A-AS took to affect Noth4, p-Akt and p-STAT3 is not clear and needs more efforts in the future.

## Data AvailabilityStatement

The datasets presented in this study can be found in online repositories. The names of the repository/repositories and accession number(s) can be found below: GSE182998, GSM5548426-29.

## Ethics Statement

The animal study was reviewed and approved by Tianjin Medical University Animal Care and Use Committee.

## Author Contributions

KZ and ZL contributed to conception and design of the study. GL and XL performed the laboratory studies. YZ and KZ carried out data analyses. KZ contributed to drafting the manuscript. All authors contributed to the article and approved the submitted version.

## Funding

This work was supported by grants from the Science & Technology Development Fund of the Tianjin Education Commission for Higher Education (2017KJ227).

## Conflict of Interest

The authors declare that the research was conducted in the absence of any commercial or financial relationships that could be construed as a potential conflict of interest.

## Publisher’s Note

All claims expressed in this article are solely those of the authors and do not necessarily represent those of their affiliated organizations, or those of the publisher, the editors and the reviewers. Any product that may be evaluated in this article, or claim that may be made by its manufacturer, is not guaranteed or endorsed by the publisher.
