# Supplementary material for: LncRNA PPM1A-AS Regulate Tumor Development Through Multiple Signal Pathways in T-Cell Acute Lymphoblastic Leukemia
Source: Front Oncol. 2021 Oct 21;11:761205. doi: 10.3389/fonc.2021.761205 (PMC8567141; doi:10.3389/fonc.2021.761205)

Supplemental Table 1. The list of data related to figure 1A

| GSM3004624 TALL_JS_10 |
| --- |
| GSM3004625 TALL_JS_11 |
| GSM3004626 TALL_JS_13 |
| GSM3004629 TALL_JS_23 |
| GSM3004630 TALL_JS_26 |
| GSM3004633 TALL_JS_37 |
| GSM1399182 Primary T-ALL sample_1 |
| GSM1399183 Primary T-ALL sample_2 |
| GSM1399184 Primary T-ALL sample_3 |
| GSM1399185 Primary T-ALL sample_4 |
| GSM1399180 Human whole thymus extract_1 |
| GSM1399181 Human whole thymus extract_2 |

Supplemental Figure 1. knockdown and overexpression of PPM1A-AS in MOLT4 cells. (A) The efficiency of PPM1A-AS shRNAs in MOLT4 cell line. (B) CCK-8 analyses of the proliferation rates of MOLT4 cells infected with control or PPM1A-AS-knockdown lentivirus. (C) EdU analyses of the proliferative ability of MOLT4 cells infected with control or PPM1A-AS-knockdown lentivirus. Left panel: representative images; right panel: average percentage of EdU^+^ cells counted in each field. Scale bar, 100μm. (D) The efficiency of PPM1A-AS overexpression in MOLT4 cell line. (E) CCK-8 analyses of the proliferation rates of MOLT4 cells infected with control or PPM1A-AS-overexpression lentivirus. (F) EdU analyses of the proliferative ability of MOLT4 cells infected with control or PPM1A-AS-overexpression lentivirus. Left panel: representative images; right panel: average percentage of EdU^+^ cells counted in each field. Scale bar, 100μm.


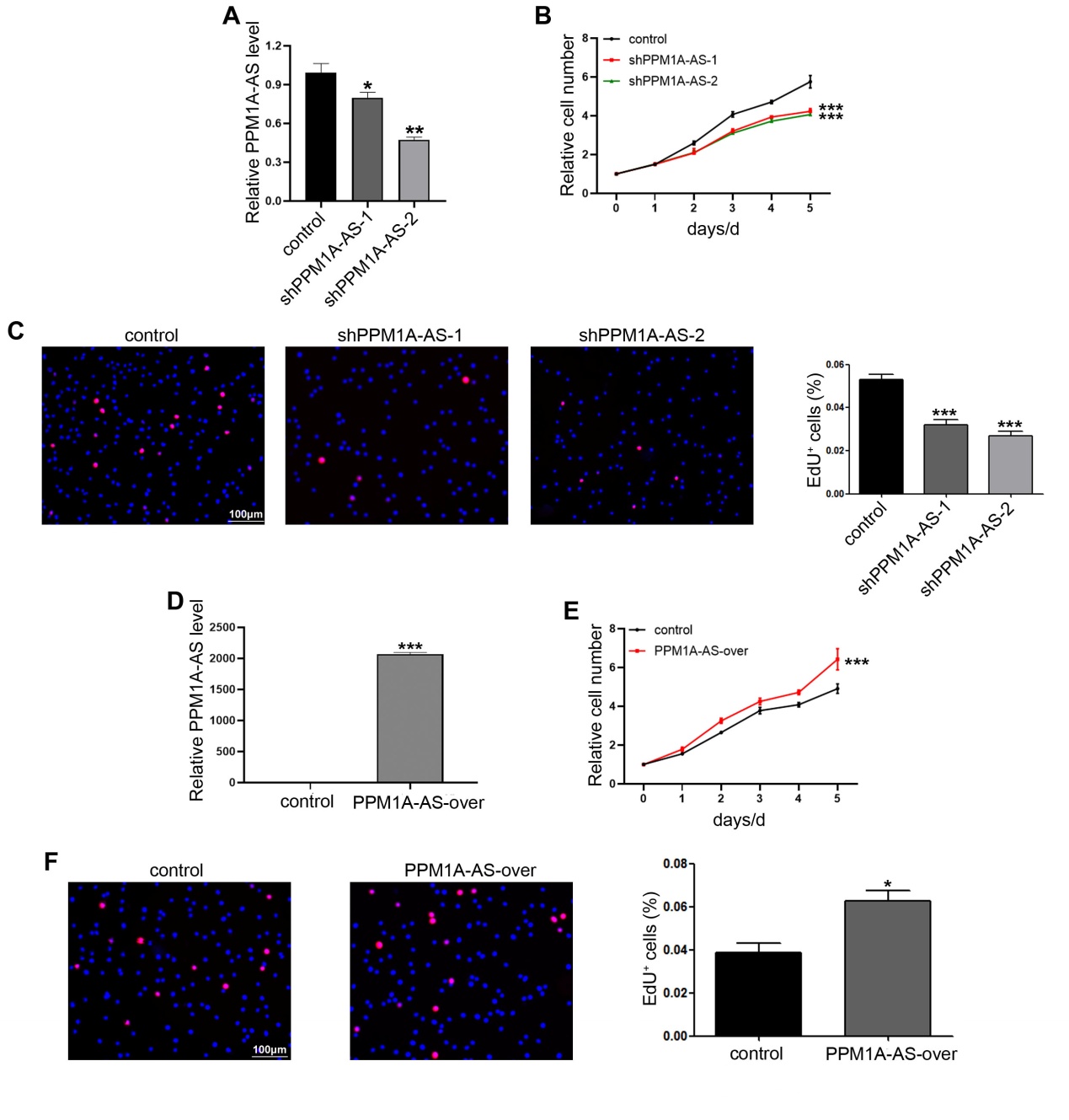

Supplement: Supplementary file 1 [file DataSheet_1.docx]
